# Cell Phone Use Habits Among the Spanish Population: Contribution of Applications to Problematic Use

**DOI:** 10.3389/fpsyt.2019.00883

**Published:** 2019-12-17

**Authors:** José De-Sola, Gabriel Rubio, Hernán Talledo, Luis Pistoni, Henk Van Riesen, Fernando Rodríguez de Fonseca

**Affiliations:** ^1^Departamento de Psicobiología, Facultad de Psicología, Universidad Complutense de Madrid Somosaguas Campus, Madrid, Spain; ^2^DE SALUD PSICÓLOGOS, Centro de Psicología y Psicoterapia, Madrid, Spain; ^3^Departamento de Psiquiatria, Universidad Complutense de Madrid, Instituto de investigación i+12, Red de trastornos adictivos, Hospital Universitario 12 de Octubre de Madrid, Madrid, Spain; ^4^Departamento de Comunicación y Marketing, Universidad Peruana de Ciencias Aplicadas, Lima, Peru; ^5^ODEC, Madrid, Spain; ^6^DYNATA, RESEARCH NOW/SSI, Madrid, Spain; ^7^Unidad de Gestión Clínica de Salud Mental, Instituto de Investigación Biomédica de Málaga—IBIMA, Hospital Regional Universitario de Málaga, Red de trastornos adictivos del Instituto de Salud Carlos III de Madrid, Malaga, Spain

**Keywords:** problem phone use, cell phone use, mobile phone dependence, problematic phone use, social media engagement, mobile phone dependence

## Abstract

Mobile phone abuse may be associated with health problems as well as with interferences in daily life. However, beyond the mobile as a device, the contributions of specific utilities and applications to the problematic mobile phone use remains to be analyzed. To address this important question we conducted 1,126 online interviews in Spain with participants aged 16 to 65 who are representative of the general population. The aim of the study was to analyze the patterns and differences of cell phone use based on habitual use, abuse, and problematic use, considering the most frequently used utilities and applications. Additional variables used were personal perception of cell phones, intensity of use, and participant’s lifestyle with regard to entertainment and the maintenance of healthy habits. Further, we aimed to analyze difference between problematic and non-problematic mobile phone users in the utilization of applications, controlling for additional variables such as age, gender, educational level, consumption of tobacco and alcohol and illegal drugs. Results show that problematic use is consistent with the self-perception of abuse and is related to internet browsing, social media, music, and mobile games; particular applications of interest include Facebook, music applications, and Twitter. Furthermore, among problematic users, a pattern of interference with other activities or inappropriate use in certain contexts is observed, with a differential pattern of entertainment and abandonment of healthy habits. Finally, two binary logistic regression analyses demonstrated that beyond the cell phones themselves, specific utilities and applications such as browsing, chatting, or downloading and listening to music contribute to the differences between problematic and non-problematic users. Specifically, the use of Facebook, Twitter, and music applications have the greatest power to discriminate between the two types of users.

## Introduction

It is by now a fact that cell phones have gone from being a new and expensive instrument for voice communication whose use was limited to contexts of wealth to a comparatively inexpensive mode of communication extended to practically all areas of life, with particularly high use among children and adolescents and for which voice and spoken communication has become a secondary use. In effect, cell phones have evolved in recent years toward the most sophisticated utilities, with text and applications having the greatest weight. According to the last annual market report on mobile phones (1), 66% of the world’s population already has a cell phone. Spain has the highest rate of penetration of any country; 88% of Spaniards possess a cell phone, and for 94.6% of Spaniards, the cell phone is the device most frequently used to access the internet. Meanwhile, more than one in four users in Spain use only a cell phone, rather than a computer, and 61% check their cell phone as soon as they get up. Similarly, daily cell phone use in Spain is 2.34 h, *versus* 1.19 h for tablets. Watching videos, using instant messaging apps such as WhatsApp, browsing the internet, and checking social networks are the most common activities, constituting the main means of accessing the web in 92.8% of cases in Spain. In general, young people in Spain dedicate far more time to their cell phones than to any other device (54%), the cell phone being present at all moments of the day; cell phones are used both when one is inactive and when one is doing other things such as working, watching television, eating, spending time with friends, or while shopping or crossing the street. This constant use of cell phones speaks to their significant power to interfere, sometimes dangerously, in everyday activities that require special attention.

Thus, today, one cannot be in the world without a cell phone; it is more than a mode of communication, it represents a link with the current social environment and to keep relationships of strong dependence (2). From this perspective, expressions of anxiety (3) and dependency or loss of control over cell phones make sense, these being related to, among other aspects, a significant dependence on the environment (4–8). Thus problematic use of the mobile phone could be defined as an inability to regulate one’s use of the mobile phone, which eventually involves negative consequences in daily life (9). Problematic mobile phone use interact with everyday activities (10, 11), specifically through the time invested on text messaging (12) or social networks, as it is the specific case of Facebook (13). Paradoxically, and from a clinical point of view, this revolution in communication, the expression of a new era and social trend can, in many cases, interfere with social contact rather than improve it (9).

In general, the innate need for security, self-esteem, and social belonging have made the cell phone an essential instrument that can nonetheless lead to dependency, a sense of loneliness, urgency of use, and craving when the device is not available or cannot be used (14), as well as the compulsive use of applications (15, 16) and searching for new sensations and distraction to overcome boredom (17, 18). In fact, there is an ever greater use and evaluation in research, using specific scales, of the term “nomophobia,” which is defined as the fear of being without one’s cell phone or without coverage at any given time (19, 20).

In effect, although the internet was initially the technological addiction par excellence, the cell phone soon emerged in parallel as a source of problematic behaviors, which have intensified with the appearance and evolution of smartphone devices (21, 22). Logically, this situation leads one to consider that it is not the cell phone as such that is problematic but rather the utilities and applications it offers, which align strongly with current demands and needs. Hence, the rapid evolution and penetration of smartphones, which represent approximately 90% of devices in Spain (1), could explain the enormous importance that the cell phone has acquired as a source of problematic behaviors that are strongly linked to the growing number of applications or “apps” offered by smartphones.

Specifically, the cell phone involves a problem related to anxiety around interactions that focus on instant messaging (23) and that translates into patterns of impulsive lack of control (24) with high levels of social anxiety, in which social contact without risks is sought out (25, 26). Moreover, it makes sense for individuals with anxiety and insecurities to seek approval and security from social networks, (27), mainly by sharing photographs and “selfies” (28), which explains why the abuse of Facebook can lead to behavioral problems, with reduced hours of sleep (13), substance use (29), and materialism or the need to have expensive brands and the latest devices (30, 31). From this perspective, it is logical that, for some time, the cell phone has been regarded as one of the biggest addictions of this century (32).

This article, which is part of a broader study, seeks to analyze patterns of cell phone use among the Spanish population, considering the everyday use, abuse, and problematic use that we have elsewhere considered a behavior comparable to addiction (33). Also considered are the personal perceptions of users as well as their lifestyles regarding entertainment and healthy habits, in addition to, specifically, the utilities or applications that have the greatest ability to explain problematic use beyond the overall consideration of the cell phone as a problematic instrument. The principal questions of this research were:

- Are there significant and relevant differences among mobile users in relation to age, gender, educational level, consumption of tobacco and alcohol and illegal drugs?
- Are there significant differences between problematic and no problematic users in the use of utilities and specific mobile applications?
- Problematic phone use could be explained by the use of specific utilities and mobile applications?

In effect, our basic hypothesis is to demonstrate that there are utilities and applications, not the device itself, that give rise to problems because of the pattern of use that is centered on social interaction. Under this perspective, applications determine a behavioral addiction/dependence based on new communication patterns. Secondarily the knowledge of the differences between problematic and non-problematic users as well as among sociodemographic and substance consumption variables could offer an opportunity to design future strategies of psychological intervention.

Finally, it should be noted that, for a long time, the focus has been on this problem among young people and adolescents. However, we believe that in the future, it may increasingly affect adult populations (34). Therefore, in this study, we consider the Spanish population as a whole in a wide range of ages.

## Materials and Methods

### Sample and Participants

The sample includes 1,126 respondents, representative from the Spanish population at the national level, both men and women, with an age range of 16–65 years. All the respondents had to be Spanish or legal residents in Spain, and to have at least one personnel mobile device in exclusive use.

The survey procedures automatically excluded uncompleted questionnaires, so only full respondents were finally used. The sampling was performed by using a non-probability procedure by quotas (sex and age) proportionate to the size of the Spanish population in all the Autonomous Communities (except Ceuta and Melilla), taking as a reference point the census conducted in 2014 by the National Institute of Statistics. Slightly more than half of the interviews were conducted in provincial capitals and in cities of over 100,000 inhabitants and the rest in rural areas and small towns

However to obtain information comparable to other current studies over-representation of segments of between 16 and 25 years and between 26 and 35 years was over-represented.

A little more than half of the interviewees were located in provincial capitals and cities with more than 100,000 inhabitants, while the rest were in rural areas and small urban centers. The sample average is 32.8 years of age, with a standard deviation of 11.67, and it consists of 47.7% men and 53.3% women. As their primary activity, more than half of those interviewed work (57.3%), with the remaining individuals being unemployed (20.2%), students (18.7%), and homemakers (3.8%). The educational level is high, with the majority of interviewees achieving higher education degrees (university or degrees) (63.5%), nearly one third having secondary education (30.4%), and a minority not going beyond basic or elementary education (6.1%). With regard to the consumption of drugs, 50.7% consume drugs generally, with 5.5% consuming illegal drugs (cannabis, hashish, cocaine, amphetamines, ecstasy, or others) *versus* 45.2% legal drugs (tobacco and alcohol) (**Table 1**).

**Table 1:** Sociodemographic distribution data and drug use from a sample of 1,126 participants in Spain.

Autonomous communities		Age		Scholing	
Andalusia	15,7%	16 to 25 years	40,9%	Higher education	63,5%
Aragón	2,5%	26 to 35 years	24,0%	Middle education	30,4%
Asturias	2,0%	36 to 45 years	17,0%	Basic education	6,1%
Balearic islands	1,9%	46 to 55 years	13,1%		
Canary islands	3,9%	56 to 65 years	5,0%	**Drugs consumption**	
Cantabria	1,2%				
Castilla La Mancha	3,9%	**Gender**		Use drugs	50,7%
Castilla León	4,4%	Male	47,7%	Don´t use drugs	49,3%
Catalonia	13,1%	Female	53,3%		
Extremadura	2,3%				
Galicia	5,0%			**Legal and illegal drugs**	
La Rioja	0,8%	**Main occupation**		Use legal drugs	5,5%
Madrid	26,2%	Worker	57,3%	Use legal drugs	45,2%
Murcia	2,5%	Unemployed	20,2%		
Navarra	1,1%	Student	18,7%	**Overall drugs use**	**50,7%**
Basque country	3,5%	Household duties	3,8%		
Valencia	10,0%				

### Procedure

Research was conducted through an online structured questionnaire completed by a survey and sociological research company that used its database of 151,170 people in Spain, between January and December 2014. The questionnaire was piloted in paper format with ten people, who were later excluded from the final sample. Each participant received a link allowing them to access a platform on which the interview was conducted using the online survey program SSI Web version 6.8 from Sawtooth Software. Participants could pause and return to the survey whenever they so desired, and the link became inactive once the survey was completed. All participants had to have their own cell phone which was evaluated through an initial filter question that conditioned the continuation of the interview.

Emailed links allowed each participant to access a platform from which the interview would begin through the survey software SSI Web version 6.8 by Sawtooth Software. It could be stopped to go back to the interview when necessary, and this link was disabled once the questionnaire was completed. All participants had to have their own mobile phone, which was assessed using a first initial filter questionnaire.

### Data Analysis

Versions 23 and 24 of the statistical package SPSS (IBM, Armonk, NY, USA) were used to perform analyses by frequencies and means, crossed with sociodemographic data on drug consumption and types of users in the Mobile Phone Problem Use Scale (MPPUS). In all cases, the statistical differences were calculated through difference of means tests (Student’s t) and difference of proportions tests. Correlational measures were also used, such as Kendall’s tau statistic and Pearson’s product-moment correlation with continuous variables (specifically, hours dedicated to cell phone use daily and number of contacts with whom contact is maintained using the cell phone). The ordinal questions for self assessment with original response ranges from 1 to 5 were reclassified in three categories (“a lot, average, or low” or “high, medium, and low”) to facilitate analysis, and age was categorized in 10‐year intervals.

The results are analyzed in relation to the age, gender, and educational level of the interviewee, consumption of substances both legal (tobacco and alcohol) and illegal (cannabis, hashish, cocaine, amphetamines, ecstasy and others), as well as with regard to the problem as measured by the Mobile Phone Problematic Use Scale (MPPUS). We established the criteria for the levels of use based on the four categories of users established by Chow et al. (35): Occasional, Habitual, At Risk, and Problematic.

In addition two binary logistic regression analyses were also conducted to discriminate between normal users (occasional and habitual users) and those with difficulties (at risk and problematic users) as a dependent variable, using the four categories mentioned above. Independent variables consisted of the most frequent cell phone uses and utilities and the specific applications that were considered essential. In all cases, the maximum admissible range of significance was 5% confidence.

### Instruments

A structured questionnaire was used that analyzes the use of devices through the perceived level of use, hours of daily use, number of contacts on the cell phone, most frequently used utilities and applications, perceived positive and negative aspects, places and times of use, most common forms of entertainment, and the maintenance of healthy habits.

The MPPUS, designed and validated by Bianchi and Phillips (36) with a sample aged 18 to 85 years, originally comprised 27 Likert-type items ranging from 1 (not at all true) to 10 (completely true). Total scores on the scale ranged from 27 to 270 points, although in our case, looking at the Spanish population as a whole, we used the adaptation developed by Lopez-Fernandez et al., (37) among adolescents, in which the original 27 items are reduced to 26; thus, in our case the maximum score is 260 points. In this sense, and despite the time that has elapsed, the MPPUS continues to be an instrument of reference, one validated and backed by a multitude of studies. Although it was initially designed for an adult population, over the years, it has been adapted for concrete groups, as in the case of the Mobile Phone Addiction Index (MPAI) for American adolescents and youth 14 to 28 years of age (17, 18); the MPPUSA, the aforementioned adaptation of the MPPUS for Spanish and English youth (37, 38); the MPPUS-10 for Swiss adolescents 12 to 17 years of age, in which the original scale is reduced to 10 items (39); a version for university students in Tehran (40); one for Japanese youth 18 to 25 years of age (41); and one for the German population 18 to 46 years of age, in which the original Likert scale is adjusted to a scale of 1 to 5 points (42). As published before, the internal reliability and consistency analysis was performed with a total Cronbach’s alpha, both for items and for averages. In general terms, the MPPUS displays good internal consistency in our sample (α = 0.939). Based on the analysis by items, none of the cases is shown to have values below 0.935, with a correlation range of 0.940 to 0.935 (see 33).

### Ethics Statement

The study and protocols for recruitment were approved by the Ethics Committee of the Hospital Regional Universitario de Málaga and were therefore conducted in accordance with the Declaration of Helsinki (seventh revision in 2013, Fortaleza, Brazil).

## Results

### Perception and Use of Cell Phones

In general terms, a large share of interviewees believe they abuse their cell phones and that they use them excessively (69.6%). More than half never turn their cell phones off before going to bed (65.9%).

Women report excessive use of their cell phones (73.7%) more than men do (65.0%) (p = 0.0017), as do both, young people 16 to 25 years of age and drug users (legal and illegal) compared to the total mean score of the sample. In contrast, users with a basic educational level demonstrate a significantly lower awareness of abuse.

On average, cell phone use is nearly 3 h per day, and the average number of contacts maintained with the device is nearly fourteen. However, the greatest investment of time is seen among young people 16 to 25 years of age, women, and users with basic education. Significant differences do not exist with regard to the number of friends with whom contact is maintained (**Table 2**).

**Table 2:** Mobile use and perception of use.

Total sample	Subpopulations with statistical differences *vs.* total sample (≤ 0,05)
Self-perception of excessive use—69,6%	-16 to 25 years—80,5% (p = 00001) -Basic education—55,1% (p = 0,0174) -Drug users (legal and illegal drugs)—75,3% (p = 0,0115)
Never turn the cell phone off before going to bed—65,9%	No differences
Daily use in hours—mean = 2,8 (SD = 2,31)	-16 to 25 years (mean = 3,51, SD = 2,46) (t = 5.3768; p = 0.0001) -Female (mean = 3,16, SD = 2,53) (t = 2.8988; p = 0,0038) -Basic education (mean = 3,48, SD = 2,78) (t = 2.3094; p = 0.0211)
Number of friends with whom contact is maintained Mean = 13,5 (SD = 11,32)	No differences

### Specific Cell Phone Uses and Applications

In general terms cell phones are primarily used to chat (91.8%), to talk (74.2%), to browse the internet (71%), to take photos (61.9%), to connect to social networks (53%), to send emails (43.1%), for fun in general (35.3%), to download or listen to music (29.7%), or to play mobile games (25.1%) (**Table 3**).

**Table 3:** General and main use of the mobile—total sample.

General use of the mobile—total sample	Subpopulations with statistical differences *vs.* total sample (≤ 0,05)
To chat—91,8%	-16 to 25 years (94,6%) (p = 0,034)
To talk—74,2%	-46 to 55 years (83,7%) (p = 0,004) -56 to 65 years (98,2%) (p = 0,000)
To browse the internet—71,0%	-16 to 25 years (77,0%) (p = 0,011) -26 to 35 years (80,8%) (p = 0,000)
To take photos—61,9%	No differences
To connect to social networks—53%	-16 to 25 years (66,8%) (p = 0,000)
To send mails—43,1%	-16 to 25 years (48,8%) (p = 0,036)
For fun—35,3%	-16 to 25 years (46,9%) (p = 0,000) -Illegal drug users (50,0%) (p = 0,023)
To download or listen to music—29,7%	-16 a 25 años (41,4%) (p = 0,000) -Illegal drug users (50,0%) (p = 0,002)
To play mobile games—25,1%	-26 to 35 years (31,4%) (p = 0,004) -Illegal drug users (43,5%) (p = 0,004)
**Main use of the mobile—total sample**	
To chat—61,4%	-16 to 25 years (69,9%) (p = 001) -Female (68,2%) (p = 0,005)
To talk—13,9%	-46 to 55 years (30,6%) (p = 0,000) -56 to 65 years (44,6%) (p = 0,000)

Talking significantly characterizes users over 45 years of age. The other utilities have greater relevance and use among those under 35 years of age compared to the total sample, as in the case of internet browsing, chatting, social networks, or mobile games. Specifically, using cell phones to download or listen to music, and for fun in general is more relevant among young people aged 16 to 25. However, the significant differences compared with the total sample are the coexistence of illegal drug use with playing mobile games, downloading or listening to music, and using the cell phone only for fun (**Table 3**).

Nevertheless, “chatting” is by far considered the main use (61.4%), basically among young users (from 16 to 25 years of age) and females, ranked far above talking (13.9%) in older subpopulations above 46 years. These proportions demonstrate the current important weight of written communication on devices (**Table 3**).

Consistently (see **Table 4**), the specific applications considered irreplaceable are WhatsApp (88.5%), the camera (58.5%), internet (57.9%), email (48.9%), Facebook (35.7%), music applications (28.5%), Twitter (20.4%), SMS (13.7%), and mobile games (10.4%). Specifically WhatsApp is considered more irreplaceable among female users, while Facebook, Twitter, and music applications are the most significantly preferable applications under age 25. Illegal drugs users show also significative preferences in considering music applications as irreplaceable. On the other hand SMS is only considered among users over 46 years (**Table 5**). It is interesting to consider that obsolescence of technologies, and rapid turnover of applications might be reflected in the different age groups.

**Table 4:** Applications considered irreplaceable.

Total sample	Subpopulations with statistical differences *vs.* total sample (≤ 0,05)
WhatsApp—88,5%	–Female (91,8%) (p = 0,023)
Photo camera—58,5%	No differences
Internet—57,9%	–Male (63,3%) (p = 0,033)
Mail—48,9%	No differences
Facebook—35,7%	–16 to 25 years (42,9%) (p = 0,008)
Music applications—28,5%	–16 to 25 years (41,4%) (p = 0,000) –Illegal drug users (41,9%) (p = 0,033)
Twitter—20,4%	–16 to 25 years (32,0%) (p = 0,000)
SMS—13,7%	–46 to 55 years (21,4%) (p = 0,028) –56 to 65 years (33,9%) (p = 0,002)
Mobile video-games—10,4%	No differences

**Table 5:** Positive and negative aspects of cell phones—total sample.

Positive aspects—total sample	Subpopulations with statistical differences *vs.* total sample (≤0,05)
To remain connected and in touch—80,3%	No differences
Stay informed—42,4%	-16 to 25 years (51,5%) (p = 0,001) -Male (50,1%) (p = 0,004)
Be entertained—33,1%	-16 to 25 years (38,9%) (p = 0,028) -Illegal drug users (48,4%) (p = 0,018)
Integration in groups of friends—16,0%	No differences
Satisfaction of using it—9,2%	-Basic education (26,1%) (p = 0,002)
Alleviate feelings of loneliness—5,0%	No differences
Alleviate anxiety—4,4%	No differences
**Negative aspects—total sample**	**Subpopulations with statistical differences vs. total simple (≤ 0,05)**
Feeling obliged to always remain connected—31,8%	No differences
Being prevented from doing other things—31,4%	No differences
Needing to have a device—25%	No differences
Feeling that one cannot be without the mobile—24,6%	No differences
Interfering with sleep or other activities—15,8%	-16 to 25 years (23,7%) (p = 0,000)
Not being able to disconnect the cell phone off—14,1%	-16 to 25 years (18,5%) (p = 0,033)
Produces worry and anxiety—11,0%	-16 to 25 years (15,4%) (p = 0,021)

### Positive and Negative Aspects of Cell Phones

Cell phones are primarily viewed positively and appreciated because they provide the possibility to remain connected and in touch (80.3%), stay informed (42.4%), be entertained (33.1%), facilitate integration in groups of friends (16%), or simply offer the satisfaction of using them (9.2%). They also serve as a resource to alleviate feelings of loneliness (5%) and anxiety (4.4%). Specifically, compared to the total users, the advantage of staying informed stands out significantly among young people aged 16 to 25, as does the use for mere entertainment. However, there are differences regarding educational level; those with basic education emphasize more positively over the other items the simple satisfaction of using cell phones without a concrete objective, which is similar to the findings for illegal drug users, where entertainment for its own sake is an advantage emphasized significantly compared to the total (**Table 5**).

In contrast, the negative aspects indicated are feeling obliged to always remain connected (31.8%), being prevented from doing other things (31.4%), needing to have a device (25%), feeling that one cannot be without the cell phone (24.6%), the cell phone interfering with sleep or other activities (15.8%), not being able to disconnect or turn the cell phone off (14.1%), and the cell phone producing worry and anxiety (11%). However, the group of young people aged 16 to 25 years, compared to the total, emphasizes significantly not being able to disconnect or turn off cell phones, sacrificing hours of sleep or producing worry and anxiety (**Table 5**).

### Places and Times of Use

Cell phones are used most commonly at home (81.5%), in the street (51.3%), on public transportation (44.8%), at work (30.2%), at sites of leisure (28.9%), while doing other things (25.4%) or in the company of other people (16.3%). However, both, illegal drug users and young people aged 16 to 25 demonstrate significantly greater use on public transportation *versus* total sample (**Table 6**). Illegal drug users demonstrate significantly greater use while in company with other people (25.8%) than do non-drug users (13.2%) (p = 0.0266). That is, in these cases, there arises a pattern of interference with other activities.

**Table 6:** Places and times of use.

Total sample	Subpopulations with statistical differences *vs.* total sample (≤ 0,05)
At home—81,5%	-Unemployed (88,1%) (p = 0,007)
In the street—51,3%	No differences
On public transportation—44,8%	-16 to 25 years (61,1%) (p = 0,000) -Illegal drugs users (58,1%) (p = 0,035)
At work—30,2%	-46 to 55 years (40,4%) (p = 0,016)
At sites of leisure—28,9%	No differences
While doing other things—25,4%	No differences
In the company of other people—16,3%	No differences

### Entertainment and Healthy Habits

The forms of entertainment analyzed were going out with friends (69%), reading (65.4%), browsing on computers at home (58.9%), watching television (57%), going to movies and other shows (56.1%), playing sports (49.2%), or traveling (46.5%). Principally, there is a greater tendency toward reading, or going out with friends among users with higher educational levels compared to those with basic education, who are more likely to prefer television compared to the total sample (**Table 7**).

**Table 7:** Entertainment and healthy habits—total sample.

Usual entertainments—total sample	Subpopulations with statistical differences *vs.* total sample (≤0,05)
Going out with friends—69,0%	–16 to 25 years (76,4%) (p = 0,002) –Female (76,0%) (p = 0,002) –Higher education (73,8%) (p = 0,024)
Reading—65,4%	–Female (78,4%) (p = 0,000) –Higher education (71,2%) (p = 0,008)
Browsing on computers at home—58,9%	–16 to 25 years (67,2%) (p = 0,002) –Male (66,0%) (p = 0,005)
Watching TV—57,0%	–Basic education (69,6%) (p = 0,026)
Movies and other shows—56,1%	–16 to 25 years (63,6%) (p = 0,005)
Playing sports—49,2%	–Male (59,1%) (p = 0,000)
Traveling—46,5%	–Higher education (53,0%) (p = 0,006)
**Healthy habits—total sample**	**Subpopulations with statistical differences vs. total sample (≤ 0,05)**
Cleanliness and personal hygiene—81,8%	-Female (85,8%) (p = 0,027)
Doing fun or agreeable things—67,0%	No differences
Going outside, sun tanning, and walks—65,7%	No differences
Maintaining a good group of friends—62,1%	–Higher education (66,7%) (p = 0,041)
Watching one’s diet—59,9%	–Higher education (64,8%) (p = 0,032)
Getting enough sleep—58,7%	No differences
Playing sports—54,0%	–Illegal drugs users (40,3%) (p = 0,029)
Doing things one enjoys—52,5%	No differences
Healthy relationships with the opposite gender—50,8%	–26 to 35 years (60,1%) (p = 0,005) –Higher education (56,8%) (p = 0,011)
Concern for physical appearance—44,5%	–Female (49,9%) (p = 0,030)
Self care—41,8%	–Higher education (46,6) (p = 0,041) –Basic education (29,0%) (p = 0,022) –Female (47,2%) (p = 0,030)
Periodic medical checkups—32,7%	–Female (37,8%) (p = 0;033) -46 to 55 years (47,6%) (p = 0,000) -56 to 65 years (48,2%) (p = 0,022)

Healthy habits considered are cleanliness and personal hygiene (81.8%); doing fun or agreeable things (67%); going outside, sun tanning, and walks (65.7%); maintaining a good group of friends (62.1%), watching one’s diet (59.9%); getting enough sleep (58.7%); playing sports (54%); doing things one enjoys (52.5%); maintaining healthy relationships with the opposite gender (50.8%); concern for physical appearance (44.5%); self care (41.8%) and getting periodic medical checkups (32.7%).

Habits particularly prominently associated with a high cultural level are the attention paid to watching one’s diet, healthy relationships with the opposite gender, having a good group of friends, or self care in general. Similarly, women also express greater periodic medical checkups, more concern for physical appearance, cleanliness, and hygiene as well as self care in general, as reflected in significant differences compared to the total sample. However, we find lack of personal care, as in the specific case of sports and physical exercise among illegal drug users, as well as specific personal self care among users that only reached basic education degrees (**Table 7**).

### Problematic Cell Phone Use

As discussed, to evaluate problematic cell phone use, we used the MPPUS by Bianchi and Phillips (36) in its Spanish adaptation by Lopez-Fernandez et al. (37). The profiles of cell phone users were obtained using the criteria by Chow et al. (35), which establish four categories of users (occasional, habitual or regular, at risk, and problematic). In another article (33), we have indicated that there is a 15.4% prevalence rate of abuse (at risk users), while problematic use stands at 5.1%, the sum of both being 20.5%, a group we will call “users with difficulties.” In other words, for simplification and subsequent analysis, we have reclassified occasional and habitual users as “normal users” and at risk and problematic users as “users with difficulties”.

With these two categories, we will see that users with difficulties demonstrate a significantly greater perception of cell phone abuse compared to normal users, with the correlation between the total MPPUS score and perception of abuse also being significant (Tau = 0.284, p = 0.000).

Regarding use of the device in general, compared to normal users, users with difficulties report using their cell phones significantly more to browse the internet, to use social networks, to download or listen to music, for fun, or to play mobile games. In contrast, although chatting is the most common utility, no significant differences exist in this category between normal users and users with difficulties, not even when this is considered the primary use above all other cell phone utilities (**Table 8**).

**Table 8:** Differences between normal users and users with difficulties in all the variables considered in this study.

	Normal users	Users with difficulties	Statistical differences *vs.* total sample (≤0,05)
Self-perception of excessive use	65%	87,4%	p = 0,000
Daily use in hours	Mean = 4,03; SD = 2,66	Mean = 2,49; SD = 2,10	t = 9.3741; p = 0,000
Number of friends with whom contact is maintained	Mean = 13,30, SD = 11,09	Mean = 14,45; SD = 12,18	t = 1.3764, p = 0.169
**General use of the mobile**			
To chat	91,9%	91,3%	p = 0.764
To browse the internet	67,7%	84,0%	p = 0,000
To connect to social networks	50,1%	64,5%	p = 0,000
To download or listen to music	26,1%	43,3%	P = 0,000
For fun	33,3%	43,3%	p = 0,006
To play video games	22,6%	35,1%	p = 0,000
**Main use of cell phones**			
To chat	60,4%	64,9%	p = 0,201
**Applications considered irreplaceable**			
Facebook	32,0%	50,0%	p = 0,000
Twitter	17, 6%	30,4%	p = 0,000
Music applications	26,2%	37,4%	p = 0,002
Email	43,0%	50,4%	p = 0,041
**Positive aspects of cell phones**			
To remain connected and in touch	81,6%	75,2%	p = 0,038
Integration in groups of friends	14,1%	23,5%	p = 0,002
Satisfaction of using it	6,5%	19,6%	p = 0,000
Alleviate feelings of loneliness	2,5%	14,8%	p = 0,000
Alleviate anxiety	2,3%	12,6%	p = 0,000
**Negative aspects of cell phones**			
Needing to have a device	20,1%	26,3%	p = 0,038
Feeling that one cannot be without the mobile	20,6%	40,2%	p = 0,000
Interfering with sleep or other activities	12,6%	27,9%	p = 0,000
Not being able to disconnect the cell phone off	11,3%	24,9%	p = 0,000
Produces worry and anxiety	8,8%	19,2%	p = 0,000
**Places and times of use**			
In the street	48,8%	61,3%	p = 0,000
On public transportation	42,7%	52,6%	p = 0,007
At sites of leisure	26,4%	38,7%	p = 0,000
While doing other things	21,5%	40,9%	p = 0,000
In the company of other people	13,2%	28,3 5	p = 0,000
**Entertainments**			
Reading	69,8%	48,3%	p = 0,000
Browsing on computers at home	60,6%	52,6%	p = 0,028
Traveling	48,0%	40,4%	p = 0,034
**Healthy habits**			
Cleanliness and personal hygiene	85,4%	68,0%	p = 0,000
Doing fun or agreeable things	69,6%	56,7%	p = 0,000
Going outside, sun tanning, and walks	68,5%	55,0%	p = 0,000
Maintaining a good group of friends	64,2%	53,7%	p = 0,004
Watching one’s diet	63,5%	46,3%	p = 0,000
Getting enough sleep	61,8%	46,7%	p = 0,000
Doing things one enjoys	55,9%	39,4%	p = 0,000
Healthy relationships with the opposite gender	52,6%	43,7%	p = 0,015
Self care	44,8%	30,3%	p = 0,000
Periodic medical checkups	34,5%	25,5%	p = 0,006
**Total users**	**895**	**231**	

Specific applications that stand out significantly among users with difficulties compared with normal users are Facebook, music, and Twitter, with significantly greater use of email among normal users compared to users with difficulties. Thus, although WhatsApp is the most widely used application, problematic use is more circumscribed to Facebook, music applications, and Twitter. This finding leads us to hypothesize that it is primarily applications that have important weight in terms of instant gratification, whether positive or negative, through the search for immediate satisfaction, or fleeing from dysphoria that end up producing the greatest dependency on and problems with cell phones.

While the positive aspects that stand out significantly among normal users compared to users with difficulties are being connected and in touch, in contrast, the latter significantly emphasize being integrated in groups of friends, satisfaction of use, alleviation of feelings of loneliness, or the cell phone as a resource to calm and remove anxiety.

Regarding negative aspects, while normal users significantly emphasize the need they feel to have a device compared to users with difficulties, users with difficulties significantly emphasize not being able to be without the device compared to normal users, the cell phone taking away hours of sleep and other activities, the impossibility of disconnecting or turning it off, and the cell phone causing worry and anxiety.

Regarding places and times of use, users with difficulties use cell phones in the street significantly more than normal users, as well as on public transportation, at leisure sites, while doing other things or when they are with people and in company. That is, there is a pattern of interference with other activities for these users.

Regarding entertainment, normal users differ from users with difficulties in reading, browsing on computers at home, and enjoyment of traveling. In the case of browsing, the differences would suggest use for leisure combined more with computers at home *versus* an excessive focus on the cell phone in different places and situations.

Similarly, there is a significant deficit in healthy habits among users with difficulties *versus* normal users, as in the case of maintenance of hygiene and personal cleanliness, doing agreeable and fun things, going outside and taking walks, having a good group of friends, watching one’s diet, getting enough sleep, spending time on oneself and things one enjoys, maintaining healthy relationships with the opposite gender, self care in general, and getting periodic medical checkups.

The number of hours of daily use is also significantly greater among users with difficulties compared to normal users. In fact, a significant positive Pearson correlation is also observed between hours of use and problematic use scores on the MPPUS, both in the total sample (r = 0.364, p = 0.000) and in all age subgroups (16 to 25 years r = 0.321, p = 0.000; 26 to 35 years r = 0.243, p = 0.000; 36 to 45 years r = 0.319, p = 0.000; 46 to 55 years r = 0.399, p = 0.000; 56 to 65 years r = 0.344, p = 0.000). This finding indicates that time of use per day is clearly related to problematic cell phone use in large sectors of the population.

In contrast, no significant differences are observed regarding the number of friends with whom one stays in contact on cell phones between normal users and users with difficulties.

In another article (33), we indicate having determined through a logistic regression analysis that the number of hours of use per day and age, in a range of up to 35 years, would be the variables with the greatest predictive ability for the probability of being a problematic user. On the other hand, educational level would have an inverse predictive weight, thus constituting a “protector” against problematic use.

Additionally, based on our final objective in this article of confirming what is truly problematic regarding cell phones and going beyond its overall consideration, we carried out two logistic regression analyses with normal users and users with difficulties as explained or dependent variables, considered independently in an analysis of the most common uses (**Table 9**) and, in the other analysis, the specific cell phone applications considered essential (**Table 10**).

**Table 9:** Binary logistic regression analysis with independent variables considered—common uses and utilities of the phone.

Cox and Snell R² Nagelkerke R²				P = 0.065 P = 0.103				
Hosmer and Lemeshow	Chi = 8.996		gl = 8	P = 0.343				
							95% for O.R.	
	β	Standard E.	Wald	gl	p (sig.)	O.R.	Lower L.	Upper L.
To talk	−0.313	0.180	3.025	1	0.082	0.731	0.514	1.040
**To browse the internet**	**0.749**	**0.219**	**11.691**	**1**	0.001 **	**2.114**	**1.376**	**3.247**
**To chat in general**	**0.764**	**0.177**	**18.745**	**1**	0.000 **	**2.147**	**1.519**	**3.035**
To send messages	−0.065	0.216	0.090	1	0.764	0.937	0.614	1.431
To send email	−0.191	0.176	1.177	1	0.278	0.826	0.584	1.167
To use social media	0.186	0.186	1.005	1	0.316	1.205	0.837	1.735
To play mobile games	0.341	0.182	3.536	1	0.060	1.407	0.986	2.008
To take photos	−0.354	0.193	3.374	1	0.066	0.702	0.481	1.024
**To download or listen to music**	**0.534**	**0.181**	**8.684**	**1**	0.003 *	**1.706**	**1.196**	**2.434**
To have fun in general	−0.177	0.183	0.929	1	0.335	0.838	0.585	1.200

Cox and Snell, Nagelkerke, and Hosmer and Lemeshow coefficients are shown, as well as independent variables with beta (β) Wald and odds ratio values, and levels of significance for the 0.05 (*) and 0.01 (**) levels. The significance of bolded texts are to highlight the significative factors in the binary logistic regression.

**Table 10:** Binary logistic regression analysis with independent variables considered (applications considered essential).

Cox and Snell R² Nagelkerke R²				P = 0.048 P = 0.075				
Hosmer and Lemeshow	Chi = 15.353		gl = 7	P = 0.032				
							95% for O.R.	
	β	Standard E.	Wald	gl	p (sig.)	O.R.	Lower L.	Upper L.
Twitter	0.522	0.181	8.349	1	0.004 *	1.686	1.183	2.403
Facebook	0.729	0.165	19.509	1	0.000 **	2.073	1.500	2.866
WhatsApp	0.080	0.248	0.103	1	0.748	1.083	0.666	1.761
SMS	−0.013	0.229	0.003	1	0.954	0.987	0.630	1.547
Mobile game applications	−0.286	0.260	1.204	1	0.273	0.751	0.451	1.252
**Music applications**	**0.556**	**0.175**	**10.155**	**1**	0.001 **	**1.744**	**1.239**	**2.456**
**Email**	**−0.563**	**0.168**	**11.273**	**1**	0.001 **	**0.570**	**0.410**	**0.791**
Camera	−0.174	0.174	0.990	1	0.320	0.841	0.597	1.183

Cox and Snell, Nagelkerke, and Hosmer and Lemeshow coefficients are shown, as well as the independent variables with beta (β) Wald and odds ratio values, and levels of significance for the 0.05 (*) and 0.01 (**) levels. The significance of bolded texts are to highlight the significative factors in the binary logistic regression.

The results indicate that of all the utilities considered, those that differ significantly between normal users and users with difficulties (at both the 1 and 5% levels) are browsing the internet (p = 0.001), chatting in general (p = 0.000) and downloading or listening to music (p = 0.003), although the first two have greater significance. It is noteworthy that while playing mobile games does not reach a 5% significance level, it does maintain very close levels of significance (p = 0.060) (**Table 9**). Therefore, the utilities that specifically determine that a user has difficulties with cell phones are primarily browsing and chatting and, secondarily, downloading or listening to music.

Considering more specifically, in the second analysis, the concrete applications that are considered irreplaceable, we see that Facebook (p = 0.000), music apps (p = 0.001), and Twitter (p = 0.004) demonstrate significant differences between normal users and users with difficulties. Email, on the other hand, maintains a significant inverse relationship (p = 0.001) with users with difficulties (**Table 10**). That is, email use is inversely related to problematic cell phone use.

Thus, it is confirmed that the cell phone is not problematic in itself, but rather, browsing, chatting through instant messaging, downloading, and listening to music and using Facebook, Twitter, and music applications in particular have the greatest discriminating power for the two types of users. However, and in line with previous results, although chatting, along with other applications, defines the behavior of users with difficulties, WhatsApp as a specific application does not discriminate between both groups of users. This finding leads us to believe that the interactivity of instant messaging communication, in its problematic aspect, likely comes from a wide spectrum of applications, not specifically from WhatsApp.

## Discussion

This study analyzed diverse aspects related to cell phone use, considering use habits, personal perception, the most used utilities and applications, lifestyles related to entertainment, and healthy habits and self care.

On the one hand, our intention has been to establish a sociodemographic profile of these variables, in relation to age, gender, and educational level as well as the consumption of drugs both legal (tobacco and alcohol) and illegal (marijuana, hashish, cocaine and others), under the hypothesis that this last variable could also explain relevant differences.

On the other hand, problematic cell phone use has been quantitatively analyzed through the adaptation (33) of the Mobile Phone Problematic Use Scale by Bianchi and Phillips (36), taking into account the aforementioned variables and considering two categories of users: normal users (the sum of both occasional and habitual users) and those users with difficulties (the sum of at risk and problematic users), based on the four categories described by Chow et al. (35). Based on these categories and considering cell phone utilities and applications as independent variables, we have also sought to confirm precisely which of these would explain problematic use, under the hypothesis that problematic use is not related to the device itself but rather to specific utilities and applications. This hypothesis is not new, as trends of research have existed along these lines for some time (43, 44). In fact, the most recent research tends to analyze specific applications such as online mobile games as responsible for problematic behaviors (45, 46).

We see that, in general terms, women, young people ages 16 to 25 and users with basic educational levels use cell phones for more hours per day, and there is also a greater perception of abuse among women, young people ages 16 to 25, and drug users (legal and illegal). Conversely, users with lower educational levels demonstrate less awareness of abuse. In this sense, and although we know that self-perceptions tend to overestimate real use over problematic use (42, 44, 47–50), we cannot underestimate self-awareness of excess, which, as we have observed in this article, correlates closely with problematic behavior. Regarding educational level, Leung (17), among others, has found a relationship between low educational levels and problematic cell phone use.

On the other hand, the idea that age is initially a vulnerability factor, especially among the youngest users, is not new (36–38, 42, 51–59), although we know that problematic cell phone usage increasingly affects more population groups (34).

Thus, the perception of abuse is related to problematic use, there being a clear awareness of when reasonable limits of use are exceeded. However, the role of educational level is relevant, as there appears to be a direct relationship between this and awareness of abuse; the higher the educational level is, the greater the recognition of abuse. Conversely, users with basic educational levels do not appear as aware of or demonstrate a recognition of this, although paradoxically, they use cell phones for the greatest number of hours per day.

Among the most prominent uses of cell phones, talking is more frequent among adults beginning at age 45, while chatting, browsing the internet, social media, playing mobile games, listening to music, or simply having fun is more common among the youngest users. Additionally, it is especially relevant that playing mobile games, listening to music, or using cell phones merely for entertainment are most prominent among illegal drug users, which suggests the hypothesis that cell phones can become a resource or instrument for evasion, distraction, coping, or control of dysphoria and anxiety (10, 24, 60–63). In this sense, some studies have already noted that the problematic aspect of cell phone use is closely related to their use without concrete objectives, as a distraction, compared to use that is focused on specific tasks and objectives (43). However, apart from voice communication, chatting or sending messages, browsing online, and using social media (primarily Facebook and Twitter) are the preferred utilities, especially among young users under age 35, and are also the areas in which problematic cell phone use is primarily centered.

In contrast to these problematic aspects, the positive perceptions and opinions about cell phones include, in addition to their value as informational tools, their important role in distraction and entertainment, especially among the youngest users, those with lower educational levels and illegal drug users. In contrast to informational uses, the youngest users indicate the most negative aspects of cell phone use, such as the impossibility of disconnecting or turning off cell phones, usage reducing hours of sleep, and usage producing worry and anxiety. This finding is consistent with the fact that the largest patterns of interference of cell phone use with other activities are found among the youngest age set and among illegal drug users, these groups being, as previously noted, more aware of their high use. Traditionally, drug use has been associated with cell phone abuse in general (64, 65), although we observed above that it is linked more specifically to social media, the internet, photos, music, mobile games, or merely fun and entertainment. Along these lines, Kuss and Griffiths (29) have indicated that the use of social media is related to substance use.

Cell phone use is also related to behavioral patterns linked to personal entertainment, the use of free time, and self care and healthy habits. Thus, while users with higher educational levels focus their interests around cultural and social activities such as traveling, spending time with friends, and reading, users with lower educational levels display a greater preference for watching television at home. The same is true of self care and maintaining healthy habits, where a high educational level is again a protector of said maintenance, and the highest levels of personal neglect are found among illegal drug users. It is noteworthy that women, despite more hours of cell phone use per day and having a greater sense of abuse, also demonstrate—as with a high educational level—a greater capacity for personal care. Thus, although they may be more inclined toward greater cell phone use and social anxiety due to the importance they place on interpersonal relationships (66), women nevertheless have a greater capacity for control over other entertainment uses and personal self-care, as we have seen in this article.

Thus, in line with our initial research question (“are there significant and relevant differences among mobile users in the relation to age, gender, educational level, consumption of tobacco and alcohol and illegal drugs?”), we have found differences taking in considerations in age, gender, educational level, consumption of tobacco and alcohol and illegal drugs with respect to the self-perception of mobile phone use and the specific use of applications. This variables also influence in the subjective evaluation of positive and negative aspects of cell phones as useful devices. The data also reflects the patterns of influence and interference in the daily life when the places and times of use of the mobile phone is considered, especially regarding entertainments and healthy habits of the users.

Considering problematic cell phone use using the MPPUS, and simplifying the four original categories of users into normal users and those with difficulties, as has been indicated, the latter demonstrate greater awareness of abuse in relation to other users regarding browsing the internet, connecting to social networks, listening to or downloading music, playing mobile games, or using cell phones merely for fun. However, chatting or sending messages, even though this is one of the most preferred utilities, does not reflect significant differences. That is, problematic use is found more in connection with internet browsing, social networks, music, mobile games, and fun and entertainment on cell phones. Specifically, Facebook and Twitter and music applications have greater relevance than WhatsApp, which has led us to hypothesize that the search for immediate relief in dealing with dysphoria and worry can inspire problematic cell phone use. This hypothesis is supported by the fact that the group of users with difficulties more positively evaluate aspects of cell phones related to being integrated into groups, the simple satisfaction of use, relief from feelings of loneliness or as a way to calm or remove anxiety, while in a negative sense, they recognize not being able to be without the device or not being able to turn it off, interference with sleep and other activities, and, paradoxically, it being a source of worry and anxiety. This finding is consistent with dependence on the social environment and group norms determining self-identity and the need for belonging, especially among adolescents (7), in which the cell phone constitutes an essential vehicle for contact, which, for some authors, explains why it can become problematic or addictive (6, 32, 67).

Likewise, users with difficulties also present a pattern of greater, sometimes dangerous, interference of cell phones with other activities and demonstrate less emphasis on direct cultural and social entertainment and activities as well as a deficit in self-care and healthy habits. In the same vein, for some time, numerous studies have demonstrated that problematic cell phone use occurs at inappropriate or dangerous times and involves serious patterns of interference with sleep, especially among adolescents (10, 11, 36, 68, 69).

However, the variable that most characterizes users with difficulties is the number of hours of daily use, which also correlates with and significantly predicts problematic use according to the MPPUS across large age groups, as we saw in another article (33). In general, the relationship found between hours of use and problematic use is not new but is, rather, in accordance with other works that indicate that consumption is higher among young people and stabilizes with age (17, 36, 56, 58, 70). However, as already indicated, recent studies using automatic records appear to indicate that self-perception of the time dedicated to content is lower that what is recorded (42).

Despite the existence of references in other studies (6, 7, 71), we have not found any weight or relevance for the number of friends with whom one maintains contact on one’s cell phone. This hypothesis was always based on the notion that it could constitute a powerful indicator of the intensity of the social network with which one is connected, which logically led to the idea that, with a larger network, there would be greater dependence on one’s cell phone.

Considering the two additional questions, the existence of differences between problematic and no problematic users in the use of utilities and specific mobile applications, and the explanation of problematic phone use on the basis of specific mobile applications, our data support an affirmative answer to both of them. There are important and relevant differences between normal and problematic users and certain applications can explain better these differences than the device itself.

### Strengths and Limitations of the Present Study

The main strength of this research is the wide and representative sample of the population of Spain. However, as usual in this type of studies, assessment of variables through the use of subjective methods, such as questionnaires, presents important limitations, since it is well-known that self-perceptions in questionnaires tend to overestimate usage. Future research will be oriented to provide specific and accurate analysis incorporating more objective methodologies. Nevertheless self-awareness of excessive use is also present among problematic users, so it is difficult to objectively measure the impact of this overestimation of use. In addition, an important limitation is the rapid turnover of applications implemented in the smartphones. For instance, when the present study was designed, Instagram was starting its ascending pathway in the social networks applications ranking, but it was not included. It is reasonable to think that a more in depth analysis of applications designs and routines had to be implemented (i.e., analysis of social recognition impact such as “likes”) to gain insight on its role as contributors to the problematic use. Finally further studies under development would establish the existence or not of causal relationships by analyzing those and new psychological factors and variables that could be associated and determine the overuse.

As a conclusion, in this article, we have gone further, given our hypothesis that the cell phone as an instrument in itself does not have a problematic or addictive effect but, rather, the specific utilities and applications that characterize problematic use. Thus, through two new logistic regression analyses, we can see that, essentially, being a user with difficulties is explained by internet browsing, chatting, and listening to music, with playing mobile games also having a value very near to statistical significance. However, and based on another analysis, Facebook, Twitter, and music apps are the applications that have significant explanatory value for users with difficulties.

Accordingly, it can be confirmed that the problematic aspect of cell phones is not necessarily the device as such but, rather, browsing, chatting with instant messenger or downloading and listening to music, with Facebook, Twitter, and music applications having the greatest discriminating power. However, although chatting, among others, defines the behavior of users with difficulties, WhatsApp as a specific application does not discriminate between the two groups of users. This finding leads us to believe that the interactivity characteristic of instant messaging, in its problematic aspect, likely comes from a wide range of applications, not specifically WhatsApp as an application.

## Author Contributions

JD-S, GR and FF designed the study. HT, LP and HR collaborated with data collection and analysis. Manuscript draft was written by JD-S, GR and FR. All authors revised the draft before submission.

## Funding

This work was financed by the Network of Addictive Disorders (Red de Trastornos Adictivos), the Carlos III Health Institute (Instituto de Salud Carlos III) and EU-ERDF (Sub-programme RETICS RD12/0028/0001 and RD16/0017/0001, and Proyectos de Investigación en Salud el PI16/01689).

## Conflict of Interest

Author LP was employed by company ODEC, Paseo de la Habana, 34, Madrid 28036. Author HR was employed by company RESEARCH NOW/SSI (Now DYNATA), Paseo de Recoletos 3, 28004 Madrid.

The remaining authors declare that the research was conducted in the absence of any commercial or financial relationships that could be construed as a potential conflict of interest.
